# Telomerase immunity from bench to bedside: round one

**DOI:** 10.1186/1479-5876-5-12

**Published:** 2007-02-26

**Authors:** Xochtil Cortez-Gonzalez, Maurizio Zanetti

**Affiliations:** 1 The Laboratory of Immunology Department of Medicine and Moores Cancer Center University of California, San Diego 9500 Gilman Drive La Jolla, CA 92093-0837, USA

## Abstract

Telomerase, a reverse transcriptase primarily devoted to the elongation of telomeres in mammalian cells, is also the first bona fide common tumor antigen. In fact, telomerase is over-expressed in > 85% of tumor cells irrespective of origin and histological type. In the past seven years, there has been considerable interest in assessing telomerase as substrate for vaccination in cancer patients to induce CD8 T cell responses. Because the activation of T cells is restricted by the MHC molecules on antigen presenting cells or tumor cells, the identification of telomerase peptides immunogenic for humans is tightly linked with HLA types. To date, a handful of peptides have been identified through a variety of screening procedures, including bioinformatics prediction, in vivo immunization of HLA transgenic mice, in vitro immunization of PBMC from normal donors and cancer patients, and processing in human tumor cells. Currently, there exist putative peptides for five major HLA types (A2, A1, A3, A24 and B7). Due to the complexity of the HLA system, trials have been performed focusing on the most prevalent HLA type, HLA-A2. Here, we summarize this collective effort and highlight results obtained in Phase 1 trials including a Phase 1 trial performed at the UCSD Cancer Center.

## Background

Active immunization (vaccination) offers the greatest advantages to prevent or control disease. Applied to the control of cancer, this concept is referred to as therapeutic vaccination. In the past decade great effort was placed exerted to identify tumor associated and tumor specific antigens [1,2] and to develop efficient methods to vaccinate cancer patients [3-6]. By and large, efforts have been directed at inducing T cell mediated responses, and particularly major histocompatibility complex (MHC) Class I-restricted cytotoxic CD8 T lymphocytes. Tumor associated antigens can be clustered in major categories. Traditionally, they were regarded as onco-developmental antigens mainly carbohydrate in nature [7]. Subsequently, new families of antigens were identified. These include tissues specific antigens, i.e., antigens found principally in one type of tumor cells, e.g., melanoma cells, prostate cancer cells, pancreatic tumor cells etc. A second group of is antigens is shared by a variety of tumors such as certain oncogenes (e.g., p53 NY-ESO1, MUC.1, and Her2-neu). These shared antigens cover a larger segment of the tumor population. A third family comprises antigens that are common to most or all tumor cells irrespective of their origin and histological type. These are molecules intimately associated with cellular processes common to all tumor cells such as immortalization or survival. Finally, there are viral antigens in tumor cells that viral antigens in those cases where a viral pathogenesis is at play (e.g., HPV, HBV and EBV).

In this review article we will recapitulate the history of one such effort as it relates to the discovery and immunological characterization of the first bona fide common tumor antigen, telomerase reverse transcriptase [8]. More importantly, the emphasis will be to demonstrate in how little time a handful of laboratories around the world interested in this new antigen converted their bench studies into bedside therapeutic vaccination intervention.

### Telomerase from cell proliferation to cell immortalization and cancer

To complete the replication of chromosomal ends, cells have evolved developed a specialized reverse transcriptase called telomerase [9], which adds a repeated sequence onto the ends of newly replicated chromosomes. Telomerase is a ribonucleoprotein, which consists of a protein component (TRT) and an RNA component (TR) containing the template for synthesis of the repeat unit added onto the ends of chromosomes [10]. Telomeres, the distal ends of eukaryotic chromosomes, stabilize the chromosomes during replication [11-13]. Telomeres shorten progressively with successive cell divisions and this shortening of chromosomal ends decreases the replicative potential of cells eventually leading cells into senescence or into crisis, which results in cell death [14]. In addition to preventing the shortening of telomeres, telomerase has been shown to protect the single-stranded ends of chromosomes and may have a role in maintaining telomeres in a structure that is not recognized as DNA damage, thereby preventing activation of the cellular senescence program [15]. In turn, maintenance of a constant telomere length ensures chromosomal stability, prevents cells from aging, and confers immortality [16-18]. This rule applies to all somatic cells. Two diseases characterized by severely premature aging, progeria and Werner's syndrome, are characterized by cells that divide only a fraction of the times normal somatic cells divide due to an abnormal telomere dynamics.

The connection of telomerase with cancer is striking. On the one hand, mice lacking telomerase RNA show that telomerase activation is a key event in malignant cell transformation [19-21]. On the other hand, *in vitro *studies in human cells show that the long-term ectopic expression of telomerase in normal fibroblasts is sufficient for immortalization but not malignant transformation [22]. However, the expression of telomerase in combination with two oncogenes (SV40 T antigen and *Ras*) promotes tumor transformation in normal human epithelial and fibroblast cell lines [23]. These transformed cells form tumors in nude mice. Thus, although telomerase *per se *is not tumorigenic, it plays a direct role in oncogenesis by allowing pre-cancerous cells to proliferate continuously and become immortal. New evidence shows that a viral telomerase RNA gene encoded by the Marek's diaease virus, an oncogene in chickens, promotes tumor formation [24]. The PCR-based TRAP assay [25] reveals a striking correlation (>85%) between high telomerase activity and tumors of different histological origins and types [26,27]. In contrast to cancer cells, normal somatic cells display little or no telomerase activity [26,28].

As anticipated above, telomerase is the first *bona fide *common tumor antigen. The history of telomerase as tumor antigen began with work in the laboratory of Lee Nadler and in our own laboratory [29,30] with the identification of 9 mer peptides of the human telomerase reverse transcriptase (hTRT) that could serve as immunogens to activate CD8 T cell precursors and generate cytotoxic T lymphocytes (CTL). This will be reviewed below.

### T cell responses against tumor and the issue of tolerance

Immunologically mediated anti-tumor responses can be attributed to a variety of mechanisms and cell types. Among the latter both NK and T lymphocytes are able to deliver a cytotoxic attack to tumor cells. However, only T cells do so by recognizing a specific antigen on the target cell and they are also capable of generating memory responses after initial expansion by antigen. Among T cell responses, CD8 T lymphocytes have received the greatest attention. These cells recognize antigen peptides of discrete size (8–10 amino acids) in the context of the MHC molecule. For this reason we, like others, have concentrated on CD8 T cell responses against telomerase.

The activation of CD8 T lymphocytes requires two signals: recognition of tumor antigen peptides in association with the MHC molecule and co-stimulation, a complex series of positive signals imparted to placed upon the T cell by the antigen presenting cell (APC) (or by the tumor cell). It is well appreciated that recognition of antigen in the absence of costimulation leads to anergy of the T cells [31], a problem often encountered in T cell responses induced by vaccination in cancer patients. Given these constraints T cell activation also depends on (a) the availability of antigen in the right form and dose, (b) the function status of the APC, and (c) the spatio-temporal relation between the APC and responding CD8 T lymphocytes in secondary lymphoid organs [32].

CD8^+ ^T lymphocytes have been documented as part of a natural response to tumor antigens in patients with cancer [33-35]. Most tumor antigens, however, are cellular components, which are encoded in the genome of the individual. They are by definition self antigens. Therefore, the immune system ability to generate immunity against tumor self antigens rests on its ability to overcome self tolerance whether this be central [36] or peripheral (reviewed in [37]). Self tolerance may exert its toll on the tumor antigen-specific T cell repertoire before cancer appears and may hamper the development of efficient anti-tumor T cell responses [38]. The hypothesis that tolerance shapes the T cell repertoire directed at tumor antigens expressed even at low levels in normal tissues, leaving behind T cells with low affinity receptors for antigen, was demonstrated in mice transgenic for the human p53 tumor antigen [39,40]. Fortunately, vaccination can expand the pool of low avidity T cells and this can control tumor growth [41]. The lesson from these studies is that in all likelihood tolerance to tumor antigens is not complete and that the low avidity T cells that have escaped tolerance and constitute the "residual T cell repertoire" can be reactivated and expanded *in vivo *by suitable vaccination.

### Identification of immunogenic peptides of human telomerase

The quest for telomerase as an antigen for cancer began with a few basic questions in mind: "Are there peptides from the consensus sequence of human TRT that bind with sufficient avidity MHC molecules and is there a residual CD8 T cell repertoire against hTRT in humans?" "Can telomerase peptides generate CTL responses that kill across tumor types?" and "Is there a residual repertoire in cancer patients?" Studies were performed using as a main approach the *in vitro *generation of specific CTL starting from peripheral blood mononuclear cells (PBMC). We identified two peptides selected on the basis of high binding to the HLA-A2 molecule: ^540^ILAKFLHWL^548 ^and ^865^RLVDDFLLV^873^, since termed p540 and p865. The same p540 was identified and analyzed in depth by Vonderheide et al [29]. While both groups found that normal individuals respond to immunization with telomerase peptides, only our laboratory showed the induction of specific CTL in cancer patients. Notably, CTL generated *in vitro *against both peptides killed peptide-pulsed target cells and tumor cells in a MHC-restricted fashion (Table 1). To complete the characterization, p540 and p865 were assessed for their immunogenicity *in vivo *using HLA A2.1 transgenic mice [42], as in these mice the peripheral CD8^+ ^T cell repertoire is essentially educated on the transgenic human molecule. Both peptides induced specific CTL responses [30], although differences were noted in that p540 induced CTL in a higher proportion of cases and CTL had greater lytic activity.

**Table 1:** Cancer cells of different origin and type are killed by anti-hTRT CTL

**Cell Target**	**Tumor Origin**	**Telomerase Activity**	**HLA-A2**	**Percent Lysis**	
				*CTL p540*	*CTL p865*
				
T2+peptide		ND	Pos.	59	48
T2		ND	Neg.	11	4
MCF7	Breast	Pos.	Pos.	39	41
SKBR3		Pos.	Neg.	7	9
SW480	Colon	Pos.	Pos.	12	37
HCT011		Pos.	Neg.	9	6
H69	Lung	Pos.	Pos.	41	9
H146		Pos.	Neg.	11	5
624	Melanoma	Pos.	Pos.	48	39
1351		Pos.	Neg.	12	6
LnCap	Prostate	Pos.	Pos.	44	41
PC3		Pos.	Neg.	9	5

From ref [30].

Following these initial reports, information on the immunogenicity of telomerase peptides accumulated rapidly. To date, immunogenic peptides have been identified for five HLA class I alleles (HLA-A*0201, B*0702, A*0101, A*0301 and A*2401) (Table 2). All of these peptides were predicted from the 1132 amino acid telomerase sequence using either one or both of two MHC binding predictive algorithms (SYFPEITHI and BIMAS) [43,44]. Actual MHC binding assays refined the prediction in the majority of cases. *In vitro *immunization of PBMC has been a constant aspect of all these studies, but *in vivo *immunogenicity in HLA transgenic mice has been used in a handful of reports only in the case of HLA-A2, HLA-B7 and HLA-A24 restricted peptides (Table 3). ^51^Cr-release assay and intracellular staining for IFN-γ synthesis or ELISPOT as a marker of CD8 T cell activation. As indicated in Table 2, a variety of model tumor cells representative of different tissues (i.e., breast, prostate, lung, colon, kidney and liver) were tested. For four peptides, information also exists with relation to tetramer positivity by the responding CD8 T cells. Since tetramer positivity correlates with the CTL induction, this approach proved useful to monitor spontaneous activation of specific CD8 T cell precursors during the development of cancer. As it will be discussed below, it is also a marker of immunological response in clinical trials. To the best of our knowledge, the information discussed above and summarized in Tables 2 and 3 represents the breadth of our current understanding of the immunogenicity of human telomerase peptides.

**Table 2:** Identification, Analysis and Characterization of HLA-restricted Telomerase Peptide

**HLA allele**	**hTRT peptide**	**Sequence**	***In vivo *peptide immunization in HLA Tg mice R/T**	***In vitro *immunogenicity in PBMC**		**Tet**	**Killing of tumor cells (hTRT**^+^**HLA**^+^**)**		**Ref.**
							
				**Normal donors**	**Cancer patients**		^51^**Cr- release assay**	**IFNγ/ELISPOT**	
A*0201	p540	ILAKFLHWL	8/10	11/12	9/13	**+**	T2*, LnCap, MCF-7, H69 624, SW480	ND	[30]
	
			ND	5/5	ND	**+**	T2*,36 M, U266, IM9, SKW6.4, K029	ND	[29]
	
			ND	ND	5/6	**+**	T2*, U266, IM9, K029, SKMEL2	ND	[66]
	
			ND	ND	21/23	**+**	T2*, BJAB, LnCap	ND	[67]
	
			ND	0/12	5/37	ND	ND	**+**	[68]
	
			ND	4/20	11/14	ND	ND	**+**	[69]
	
	p865	RLVDDFLLV	7/10	7/10	5/9	**+**	T2*, LnCap, MCF-7,624, SW480	ND	[30]
	
			ND	0/12	4/37	ND	ND	**+**	[68]
	
			ND	2/20	6/14	ND	ND	**+**	[69]
	
	p572	RLFFYRKSV	ND	0/3	1/4	ND	T2*	ND	(unpublished data)
	
	pY572	YLFFYRKSV	7/7	4/6	5/8	**+**	T2*, U266, HELA-HHD	ND	[49]

B*0702	p277	RPAEEATSL	4/6	7/8	ND	ND	T2-B7*, T1-B7, JY, Jurkat	ND	[70]
	
			5/6	5/8	ND	ND	ND	ND	[70]
	
	p342	RPSFLLSSL	2/6	2/8	ND	ND	T2-B7*, T1-B7, JY, Jurkat	ND	[70]
	
			4/4	5/8	ND	ND	ND	ND	[70]
	
	p351	RPSLTGARRL	5/6	6/8	ND	ND	T2-B7*, T1-B7, JY, Jurkat, LB34, KUL68, U293T	ND	[71]
	
	p444	DPRRLVQLL	2/7	1/8	ND	ND	ND	ND	[70]
		
	p464	FVRACLRRL	4/4	3/8					
		
	p1107	LPGTTLTAL	2/4	5/8					
				
	p1123	LPSDFKTIL	7/8	10/11	2/2		T2-B7*, JY	**+**	

A*0101	p325	YAETKHFLY	ND	1/2	ND	ND	EVB-DDU*, MOU, AKR	**+**	[72]

A*0301	p973	KLFGVLRLK	ND	2/3	2/2	ND	T2-A3*, U266, SKMES1, NHL, SK-MEL-2	ND	[73]
	
				2/6	4/7	ND	ND	**+**	[69]

A*2401	p324	VYAETKHFL	2/3 (pDNA hTRT)	2/17	9/72	ND	EBV-PBMC*, KH88, MEG01, OUN1, HepG2, HuH6, HuH7	**+**	[74–76]
		
	p461	VYGFVRACL	3/3 (pDNA hTRT)	3/17	5/72	**+**	EBV-PBMC*, HepG2, HuH6, HuH7	**+**	
			
	p1088	TYVPLLGSL	3/3 (pDNA hTRT)	0/13	6/72	ND			[74–76]
		
	p845	CYGDMENKL	2/3 (pDNA hTRT)	0/13	6/72	ND			
			
	p637	DYVVGARTF	2/3 (pDNA hTRT)	0/11	9/72	ND			[76]
		
	p167	AYQVCGPPL	1/3 (pDNA hTRT)	0/11	9/72	ND			

R/T: responders/total, Tet: Tetramer staining, ND: Not Done

*, denotes target cells pulsed with corresponding peptide

**Table 3:** *In Vitro *CD8 T Cell Response against Telomerase Peptides in Cancer Patients

**HLA Allele**	**hTRT peptide**	**Tumor type**	**Killing of tumor cells**		**Tumor infiltrating lymphocytes**	**Cancer Patient *in vitro*PBMC immunization R/T**	**Ref.**
			**Autologous primary tumor cells**	**Established tumor cell lines**			

**A2**	p540	Prostate	ND	T2*, LnCap	ND	6/9 (66%)	[30]
		Melanoma	ND	T2*	ND	1/4 (25%)	(unpublished data)
		Hematological malignancies and advanced prostate	NHL	ND	ND	5/6 (83%)	[73]
		Prostate, Breast, Lung, Gastric, NHL, Liver	Prostate cancer cells	ND	ND	21/23 (91%)	[67]
		Breast	ND	ND	ND	11/14 (79%)	[69]
		Colorectal	Colorectal cancer cells	ND	ND	5/37 (13%)	[68]
	
	pY572	Prostate	ND	T2*	ND	5/8 (62%)	(unpublished data)
	
	p865	Colorectal	Colorectal cancer cells	ND	ND	4/37 (11%)	[68]
		Breast	ND	ND	ND	6/14 (43%)	[69]
		Prostate	ND	T2*	ND	3/6 (50%)	(unpublished data)
		Melanoma				2/3 (67%)	

**B7**	p1123	Prostate	ND	T2-B7*	ND	2/2 (100%)	[70]

**A3**	p973	Breast	U266, SK-MES-1, SK-MEL-2, NHL	T2-A3*,	ND	4/7 (57%)	[69]

**A24**	p1008	Liver	ND	HepG2, HuH6 and HuH7 (Hepatoma)	ND	6/72 (8%)	[76]
	p845					6/72 (8%)	
	p167					9/72(13%)	
	p461				**+**	5/72 (7%)	
	p324				ND	9/72 (13%)	
	p637					9/72 (13%)	

R/T: responders/total, ND: Not done

*, denotes target cells pulsed with corresponding peptide

Based on the foregoing, several conclusions can be made. The first is that the prerequisite that CTL against telomerase would kill tumor cells of different origin and type was verified, consistent with the idea that telomerase is a common tumor antigen. The second is that humans possess a residual CD8 T cell repertoire directed at telomerase. Surprisingly, on average it appears that the response rate in cancer patients is equal to if not higher than that of normal donors. Of note, assessing immunogenicity of telomerase peptides required the combined use of different approaches. In selected instances, the same peptide was identified by groups working independently, validating the general understanding that telomerase is indeed immunogenic in humans.

### *In vitro *studies in cancer patients

Cancer patients responded to *in vitro *immunization at a surprisingly high rate (Table 2). It was then important to answer the question: "Did the response rate differ in patients with different types of cancers? " The response in cancer patients has been assessed in four HLA types: HLA-A2, HLA-B7, HLA-A3 and HLA-A24 (Table 3). Overall, all cancer patient categories responded albeit noticeable differences were found depending on the type of cancer. For instance, patients with colorectal or liver cancer were found to respond poorly compared with to patients with prostate, breast, lung cancer or melanoma. While it is difficult to determine if this is due to technical differences among laboratories, differences in response in various HLA groups or simply differences among patients, it is clear that this issue requires further work. It should be noted, however, that irrespective of the response rate, in some of the studies CTL generated *in vitro *against telomerase lysed autologous primary cancer cells (Table 3). Of note, in liver biopsies it was found that some tetramer positive tumor infiltrating lymphocytes were found suggesting an active recruitment of telomerase-reactive CD8 T cells at the tumor site. This type of analysis should be performed more systematically whenever possible to assess the extent to which telomerase-specific immunity contributes to the general anti-tumor response that is enriched at the tumor site.

### Phase 1 trials

A synopsis of the Phase 1 trial conducted and published to date is provided in Table 4. A telomerase-specific immune response following therapeutic vaccination was first reported by Su et al [45] in patients with metastatic renal carcinoma vaccinated with autologous dendritic cells transfected with renal tumor cell derived mRNA (Table 4). In this trial, a great proportion of patients responded immunologically as determined by ELISPOT analysis and a ^51^Cr-release assay using mRNA renal tumor transfected dendritic cells as targets or stimulators respectively. Although, in this trial patients were not specifically vaccinated against telomerase, a telomerase specific response was measured, arguing for the presence of telomerase mRNA as a component of total tumor cells mRNA along with the mRNA of other tumor antigens. In four of the five subsequent Phase 1 trials, patients were immunized against the high affinity, HLA-A2 restricted p540 telomerase peptide. Each trial used a different vaccination strategy. Thus in one case, renal, and colon cancer, and melanoma cancer patients were vaccinated with p540 in incomplete Freunds' adjuvant (IFA) [46]. In a second trial, prostate and breast cancer patients were vaccinated with dendritic cells (DCs) pulsed with p540 and KLH as a source of T helper cell determinants [47]. Non-small cell lung cancer patients were vaccinated with soluble p540 in GMC-SF in conjunction with a second telomerase peptide, p613, a promiscuous HLA-DR, HLA-DQ and HLA-DP biding peptide as a source of T cell help [48]. In the trial performed at UCSD, which will be discussed in detail below, prostate cancer patients were vaccinated with autologous transgenic B lymphocytes, a new vaccine approach tested for the first time in humans in this trial, targeting the immune response against p540 and pY572 [49] (see also Table 2). Finally, Su et al [50] vaccinated metastatic prostate cancer patients with dendritic cells transfected with telomerase mRNA. As indicated in Table 4, the immunological response averaged between 40–100% in all but one trial. In the report by Brunsvig et al. [48] only 2 out of 24 patients (8%), responded to the high affinity p540 telomerase peptide. A possible explanation for this low response rate may be due to the lack of pre-selection of patients based on HLA typing. A larger number of patients (11/24) did, however, respond to the MHC Class II peptide. Collectively it appears that an immunological response after vaccination was detected in the majority of vaccines irrespective of the modality of vaccination, confirming earlier conclusions that cancer patients have a residual repertoire of telomerase specific CD8 T cell precursors that can be expanded *in vivo *by vaccination.

**Table 4:** Human Telomerase Reverse Transcriptase Clinical Trials

**Clinical Trials**	**Vaccine Type**	**Cancer type**	**HLA**	**Immune monitoring**	**Responding patients**	**Ref.**
Phase 1	Renal tumor mRNA transfected DCs	Metastatic **renal **carcinoma (stage IV)	No restriction	ELISPOT, ^51^Cr-assay	6/7 (86%)	[45]

Phase 1	p540 in IFA	Metastatic Cancer (**Renal, melanoma, colon**)	HLA-A2^+^	Tetramer staining, IFNγ secretion	7/14 (50%)	[46]

Phase 1	p540 in KLH DCs	Hormone-independent **prostate **Cancer	HLA-A2^+^	Tetramer staining, ELISPOT, Ag-specific lymphocyte proliferation assay	2/5 (40%)	[47]
		Metastatic **breast **cancer	HLA-A2^+^		2/2 (100%)	

Phase 1	Autologous transgenic B lymphocyes (p540 and pY572)	Androgen-Independent **prostate **cancer	HLA-A2^+^	Tetramer staining, Expansion of peptide-reactive CTL, ^51^Cr-assay	10/15 (67%)	[51]

Phase 1	hTRT mRNA-transfected DCs	Metastatic **prostate **cancer	No restriction	ELISPOT, ^51^Cr-assay, Ag-specific proliferation assay	8/9 (89%)	[50]

Phase 1	p540, p613 (HLA-DR, -DQ -DP) and GM-CSF	Non-small cell **lung **cancer	No restriction	^51^Cr-assay, lymphocyte proliferation assay	11/24 (46%) response to p611	[48]
			No restriction		2/24 (8%) response to p540	

R/T, Responders/Total

The phenotype of the induced CD8 T cell responses was also characterized in two trials. In one case, responding cells were found to be CD45RA^+^/CD45RO^+^/CCR7^-^/CD27^+^/CD28^+ ^corresponding to an effector memory cell phenotype [47] whereas in the other case, they were CD45RA^+^/CD45RO^-^/CCR7^-^/CD27^- ^corresponding to an effector cell phenotype [50]. Finally, consistent with the fact that all these trials were Phase 1 trials with a target patient population with advanced stage cancer, clinical responses were found in only a limited number of cases. Thus, a partial clinical response was reported for 1 of 5 prostate cancer patients vaccinated with dendritic cells pulsed with p540 plus KLH [47]. A complete response was observed in one patient with stage IIIA non-small cell lung carcinoma at the time of vaccination [48]. Interestingly, in this patient a telomerase specific T cell response in the blood could be documented only after the first booster injection but not subsequently.

Collectively, this first round of clinical trials demonstrated vaccination against telomerase is safe and that a specific immunological response can be induced *in vivo*, even though much work needs to be performed to better characterize phenotypically and functionally the T cell response one obtains *in vivo*.

### The UCSD Trial

We will summarize herein the main outcome of a Phase 1 telomerase cancer vaccine trial held between June 2003 and January 2005 at UCSD [51,52]. In this trial, we utilized a new approach to vaccination aimed at optimizing the host capacity to generate effective T cell responses by synchronizing the activation of T cells within parameters of space, time and antigen dose as discussed in (for review see [53]). The vaccine was ultimately designed to generate CD8 T cell responses against p540 and p572. We used primary B lymphocytes transgenic for non-viral DNA as a source of APC [54], an approach termed "transgenic lymphocyte immunization." This relies on the fact that B lymphocytes spontaneously internalize plasmid (p)DNA [55] an event that turns them into efficient APCs. We observed that in the first 24 hours following pDNA internalization, B lymphocytes undergo antigen synthesis and up-regulation of costimulatory molecules, making them a new form of genetically programmed APC [53]. *In vitro *studies had shown that transgenic B lymphocytes as the only source of APC activates both CD4 and CD8 T cells, albeit the latter required T cell help [56]. *In vivo *studies had shown that the intravenous (i.v.) injection of transgenic lymphocytes in small numbers is highly effective in inducing both CD4 and CD8 T cell responses [54,57,58]. The success of these studies owes in all likelihood to the fact that after i.v. injection, transgenic B lymphopcytes localize in secondary lymphoid organs, the spleen and lymph nodes. Thus, the advantage of direct transgenic lymphocyte immunization is that it genetically programmed APC to the site of immune induction (for review see [53]).

Fifteen patients with hormone resistant prostate cancer were vaccinated by i.v. injection of autologous transgenic lymphocytes. The median age was 73, with median performance status of 1, and a median pre-treatment PSA of 148. All but three patients had metastases in bone and/or lymph node. Two patients had recurrent local disease only, and one had PSA-only disease. Patients were divided into five cohorts of three patients each. Cohort 1–3 received a single injection of transgenic lymphocytes (10^4 ^– 10^6^), respectively. Cohort 4a and 4b received two injections of 5 × 10^5^transgenic lymphocytes one month apart. In cohort 4a, the second injection utilized freshly prepared transgenic lymphocytes whereas in cohort 4b the booster vaccination was performed with frozen-thawed cells. Infusions were well tolerated with no toxicity. Using a sensitive RT-PCR (1–10 positive cells/10^6 ^cells) the transgene could not be amplified from blood lymphocytes either hours, days or weeks following injection.

#### i) Transgenic lymphocyte immunization induced tetramer+ CD8 T cells

**Figure 1:**
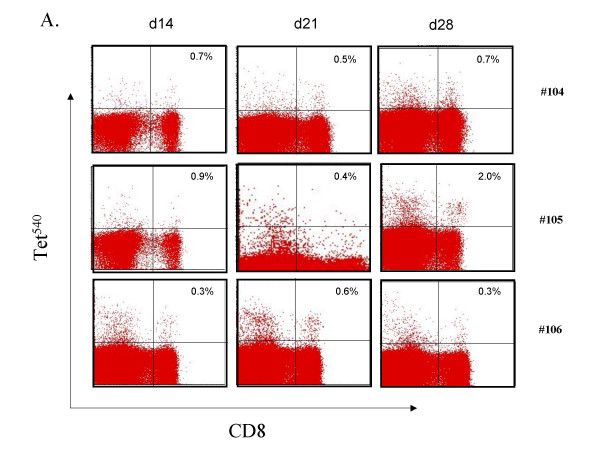
Detection of tet-positive CD8 T lymphocytes after single vaccination with transgenic B lymphocytes. (A). *Ex vivo *detection of tet^540^-positive CD8 T cell responses on day 14, 21 and 28 in vaccines of cohort 2 who received a single vaccine injection of 10^5^transgenic B lymphocytes. (B) Example of expansion of tet^540^-positive CD8 T cell responses after *in vitro *peptide restimulation in patient 107 who received a single vaccine injection of 10^6^transgenic B lymphocytes. R – restimulation in culture followed by the number of restimulations.

Tetramer staining was used to assess induction of CD8 T cell specific for hTRT p540 and pY572. None of the three patients in Cohort 1 had detectable tet^540 ^or tet^Y572^-reactive CD8 T lymphocytes in their blood when examined by FACS *ex vivo*. However, in one patient (#103) tetramer-positive CD8 T cells were rapidly expanded from PBMC of the day 56 blood draw. A response against p540 but not pY572 was visible in all three patients in Cohort 2. Tet^540 ^positive CD8 T cells were maximal on day 28 in two patients and on day 21 in the third patient (#106) (Figure 1A). All patients in Cohort 3 had detectable tet^540 ^response and to a lesser degree a tet^Y572 ^response. Restimulation of the day 56 blood draw with p540 caused rapid expansion of tet^540^-reactive CD8 T lymphocytes (Figure 1B). Thus, a single immunization with transgenic lymphocytes induced tet^540 ^CD8 T cells detectable *ex vivo *in Cohort 2 and 3. In all but two patients, peptide re-stimulation *in vitro *expanded CD8 T lymphocytes of the day 56 blood draw suggesting that immunization had likely expanded the pool of CD8 T cells specific for p540. On the other hand, no tet^Y572 ^CD8 T cells by *ex vivo *FACS staining were observed. However, tet^Y572^-reactive CD8 T lymphocytes were expanded *in vitro *by restimulation of the day 56 blood draw lymphocytes in one out of three patients in each cohort. Collectively, after single injection, tet^540 ^CD8 T cells were detected in 6/9 and tet^Y572 ^CD8 T cells in 0/9 patients. After *in vitro *restimulation on day 56, tet^540 ^CD8 T cells were expanded in 7/9 and tet^Y572 ^CD8 T cells in 3/9 patients.

**Figure 2:**
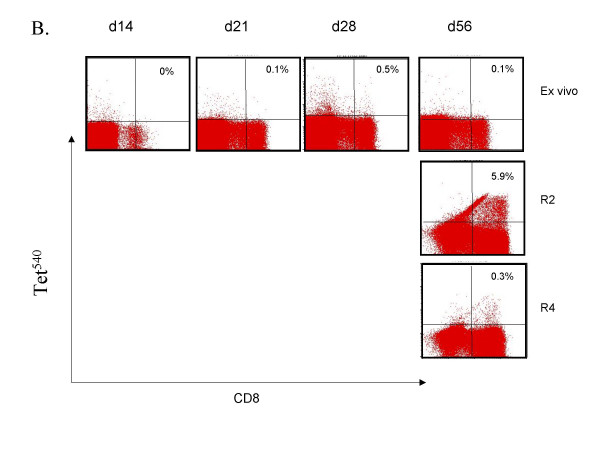
Detection of tet^540^-positive CD8 T lymphocytes after booster vaccination with transgenic B lymphocytes. (A). Tet^540^-positive CD8 T cell responses, *ex vivo *and after *in vitro *peptide restimulation on day 28 and 43, in subject #115 of cohort 4b who received an injection of freshly prepared transgenic B lymphocytes at the dose of 0.5 × 10^5 ^followed one month later by a similar dose of transgenic lymphocytes. R – restimulation in culture followed by the number of restimulations.

**Figure 3:**
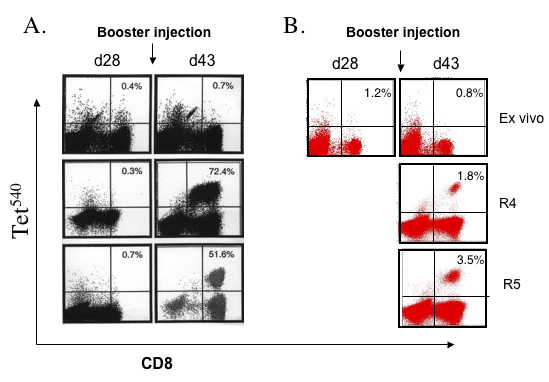
Detection of tet^572^-positive CD8 T lymphocytes after booster vaccination with transgenic B lymphocytes. (A). Tet^572^-positive CD8 T cell responses, *ex vivo *and after *in vitro *peptide restimulation, in patient #112 of cohort 4a who received two injections of transgenic freshly prepared lymphocytes at the dose of 0.5 × 10^5 ^each one month apart. (B). Detection of tet^572^-positive CD8 T cell responses, *ex vivo *and after *in vitro *peptide restimulation, in patient #115 of cohort 4b who received an injection of freshly prepared transgenic B lymphocytes at the dose of 0.5 × 10^5 ^followed one month later by a similar dose of transgenic lymphocytes.

The effect of a second injection four weeks after priming was investigated in six patients (cohort 4a and 4b). An overall increase in tetramer-positive CD8 T lymphocytes 13 days after the second injection (day 43) yielded a modest increment in the number of circulating tet^540 ^CD8 T cells in all six patients (40 ± 14/10^4 ^CD8 T cells on day 30 *vs*. 80 ± 29/10^4 ^CD8 T cells on day 43). A modest increment in tet^Y572 ^CD8 T cells occurred in 3/6 patients (26 ± 16/10^4 ^CD8 T cells on day 30 vs. 84 ± 49/10^4 ^CD8 T cells on day 43). However, when lymphocytes of the day 43 blood draw were restimulated in culture with p540 a distinct expansion was observed in two patients of cohort 4a (Figure 2A and Figure 2B). Figure 2A also shows no expansion using the day 28 blood harvest suggesting that the expansion documented 15 days after the booster injection was likely the result of the second vaccine injection. Tet^540 ^CD8 T cells could not be expanded from lymphocytes of the day 85 blood draw. The second injection had a visible effect on the expansion of tet^Y572^-positive CD8 T lymphocytes in four patients, three in cohort 4a and one in cohort 4b. The expansion of tet^Y572^-positive CD8 T lymphocytes was most consistently observed using lymphocytes of the day 85 blood draw (Figure 3A and 3B). This suggests that in the same patient there may be different *in vivo *kinetics of CD8 T lymphocytes in response to p540 and pY572 immunization. Collectively, after the second injection whereas tet^540 ^CD8 T cells were expanded in culture in 3/6 patients on day 43 and 0/6 patients and on day 85, tet^Y572 ^CD8 T cells were expanded in 4/6 patients both on day 43 and 85.

**Figure 4:**
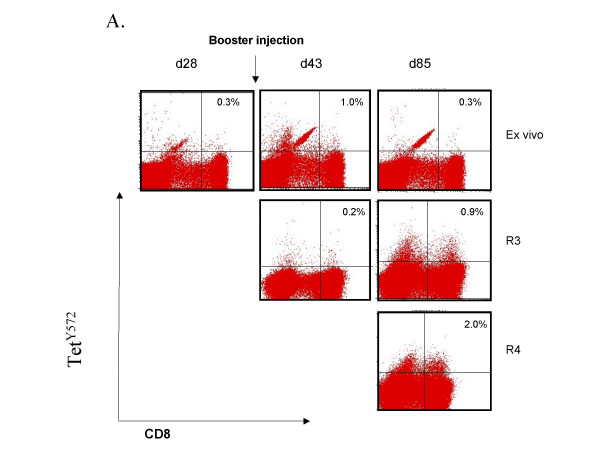
The cytotoxic activity of CD8 T cells expanded *in vitro *(R4) from lymphocytes of subject #112 of cohort 4a who received two injections of freshly prepared transgenic lymphocytes at the dose of 0.5 × 10^5 ^each one month apart. Upper panel: lysis of T2 cells with (close symbols) or without (open symbols) p540. Lower panel: lyses of MCF-7 (open symbols) or PC3 (close symbols) tumor cells. Data in both panels refer to a single experiment where the values of triplicate wells were within 5% variation.

CTL responses were sought in one patient in each cohort to probe the functionality of tetramer-positive CD8 T lymphocytes. In cohort 1, patient #103 had a small but specific lysis of T2 pulsed with p572 (42% vs. 19% on control targets) but not of T2 cells pulsed with p540. In cohorts 2 and 3, lysis of target cells pulsed with peptide was comparable to lysis of non-pulsed cells arguing for the expansion of NK cells. The two patients in cohort 4b lysed T2 target cells pulsed with p540 but not p572. In one case in cohort 4a, CTL reactive with p540 (Figure 4 upper panel) also lysed the HLA-A2^+ ^MCF7 tumor cells (Figure 4 lower panel) but not control HLA-A2^- ^PC3 cells. Although non-specific lysis was observed at the highest E:T ratio, this waned, decreasing the E:T ratio implying specific recognition of the 540 peptide in both instances.

#### ii. No untoward effects on circulating lymphocyte levels

**Figure 5:**
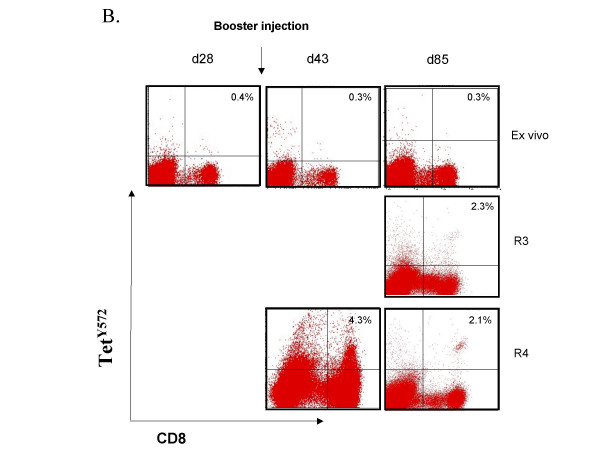
Longitudinal survey of circulating B and T lymphocytes in vaccinated patients during the short term follow-up. B lymphocytes (CD19^+^) in cohorts 1–3 (A) and in cohorts 4a and 4b (B). CD8 T lymphocytes in cohorts 1–3 (C) and in cohorts 4a and 4b (D). CD4 T lymphocytes in cohorts 1–3 (E) and in cohorts 4a and 4b (F). Data were analyzed and plotted as described in Materials and Methods, and are expressed as the % variation from pre-vaccination value for each subject.

Since telomerase activity has been reported in activated B and T lymphocytes [59-61], it was important to monitor changes in the number of circulating B and T lymphocytes longitudinally through the end of the short-term follow-up (Figure 5). The proportion of circulating B lymphocytes (CD19^+^) (panels A and B) and CD4 T lymphocytes (panels E and F) was substantially unchanged over the corresponding values of the pre-immunization time point, irrespective of the dose and number of injections, through the end of the short-term follow-up. Similarly, the proportion of circulating CD8 T lymphocytes in patients remained within a range comparable to that of the pre-immunization time point (time zero) through the end of the short term follow up period (panels C and D) and even though in patients who were given two injections a negative trend showed at the end of the observation period (panel D) even in patients who were given two injections. A non-parametric test using Spearman correlation coefficients of a global hypothesis of non-zero trend of mean CD19, CD4, or CD8 values over time after dose administration at study day 0, for cohorts 1–3 and separately for cohorts 4a-4b, each adjusted for multiple testing, yielded a non-significant result. This suggests that immunization against hTRT using transgenic lymphocytes does not affect the number of lymphocytes in the peripheral blood.

#### iii. Clinical response

No formal PSA responses were found. However, at a more accurate analysis, the PSA values distinguished three groups: In the first group (5/15) in which the PSA was considered stable the variation was < ± 25%. In the second group (6/15) the PSA value increased over the pre-treatment value but at the end of the short-term follow-up was less than double the starting value. In the third group (4/15) the PSA values doubled during the short-term follow-up.

## Conclusion

With this review article, it was our intent to provide an historical perspective of the discovery of telomerase as the first common cancer antigen, describing the steps that in quick progression took the process from the bench to the bedside. As described, the field developed quickly and the transition from the test tube to Phase 1 trials has been a rapid one.

With respect to the preclinical phase, one basic prerequisite in the successful selection of telomerase peptides was an element of empiricism. Validation of potentially immunogenic peptides depended on a number of criteria among which good binding to the MHC molecule, while necessary, was just the initial one. One important result out of the *in vitro *studies was that humans forms a residual CD8 T cell repertoire against telomerase.

Contrary to our original prediction, the residual CD8 T cell repertoire against telomerase in cancer patients was found to be conserved as in normal individuals. The fact that equal or greater expansion *in vitro *of CD8 T cells specific for telomerase peptides was found in cancer patients as compared to normal individuals indicates that peripheral tolerance does not affect CD8 T cells specific for this self tumor antigen in a way that prevents these cells from being reactivated *in vitro *by peptide stimulation. Equally unimpeded seemed the activation and expansion of CD8 T cell precursors *in vivo *as far as one can generalize from HLA-A2^+ ^cancer.

Given the widespread expression of telomerase in tumor cells of different origin and type, it ultimately it was not surprising that the same CTL could lyse tumor cells of different type and origin. Since this has been verified using CTL generated in normal individuals [29] as well as in cancer patients [30], a reasonable conclusion is that the fine specificity of the residual repertoire in cancer patients does not differ from that of normal individuals, suggesting that peripheral tolerance does not result in a preferential expansion of CD8 T cells reactive with that tumor. In other words, processing and presentation of a given telomerase peptide is fundamentally similar in different types of cancer.

Two groups have casted doubts on whether hTR p540 is processed and presented in cancer cells. Ayyoub et al [62] failed to demonstrate processing of p540 by purified human proteasome or immunoproteasome *in vitro*. In the same study these authors failed to demonstrate lysis of three hTRT-positive melanoma cell lines while the same cll lines pulsed with synthetic p540 were killed excluding an intrinsic refractoriness to lysis or defective HLA expression. In a second report Parkhurst et al [46] was unable to document specific recognition of hTR-positive melanoma and renal cell carcinoma tumor cells by vaccine-induced CTL. These authors also failed to visualize the HLA-A2-p540 complex at the cell surface of melanoma cell lines using an anti-complex antibody. However, since the affinity of this antibody is low and the minimum number of HLA-A2-p540 complexes sufficient and necessary for surface staining visualization is unknown, the significance of these negative results remains unclear. In contrast studies from a variety laboratories have doacumented lysis of tumor cells The existence of these negative reports should foster new studies to assess the reason(s) for this discrepancy among different laboratories.

Safety and potential for harmful autoimmunity were plausible concerns of telomerase vaccination in humans. An in depth discussion of this issue can be found in [8]. Telomerase is absent or undetectable in most somatic cells [16,17,28] and it becomes detectable in mitotically-active cells in normal tissues [63]. Indeed telomerase activity was documented in fractions of leukocytes enriched for B cells, T cells and monocytes [25] and in proliferating hemopoietic stem cells concomitantly [64]. Germinal center B lymphocytes express telomerase at high levels (~100 fold the levels found in naïve and memory T cells [59] and *in vitro *activation with anti-CD40 antibodies and IL-4 [59] activates telomerase in B lymphocytes. Similarly, in primary T lymphocytes telomerase activity is highly inducible by activation through CD3, with or without similar CD28 co-stimulation [65]. Thus, in both B and T lymphocytes, telomerase expression is activation-dependent and correlates with cell proliferation. Notwithstanding these considerations, experimental *in vitro *data suggested that CTL specific for the HLA-A2 restricted p540, p865 or py572 peptides do not lyse bone marrow-derived HLA-A2^+ ^CD34^+ ^cells [30], activated T cells [66] or CD40-activated B lymphocytes [49]. In line with these *in vitro *studies, it was not surprising that telomerase vaccination of cancer patients did not yield signs of autoimmune attack on normal cells. The Phase 1 trials performed to date show telomerase vaccination to be safe. In the UCSD trial, we found that the levels of circulating B and T lymphocytes did not decrease from the pre-vaccination value (Figure 5). Collectively, the existing information permits us to provisionally conclude that the risk of autoimmunity following telomerase vaccination is minimal and while continuous surveillance is appropriate, excessive concerns are not.

In summary, telomerase as a candidate cancer vaccine has been validated during the past six years. At this time, conclusions can be made with respect to issues of great fundamental interest and immunological significance. For instance, it is clear that central and peripheral tolerance against this self-antigen do not represent an obstacle to expansion of specific CD8 T cell precursors by vaccination even though the affinity of these T cells was not studied. Precursor CD8 T cells against telomerase in cancer patients can be expanded *in vivo *by various vaccine approaches. Finally, it appears that within the confines of the regimens of immunization tested so far, no untoward effects against normal cells should be expected and that the risk of autoimmunity is minimal. From only Phase 1 trials, it is premature to assess the clinical benefit of telomerase vaccination. In the future, it will be important to compare various approaches to expand the pool of telomerase specific CD8 T cells *in vivo *in cancer patients with respect to the type of CD8 T cells they expand and their clinical response.
